# Expression of high affinity folate receptor in breast cancer brain metastasis

**DOI:** 10.18632/oncotarget.4639

**Published:** 2015-06-25

**Authors:** José Pablo Leone, Rohit Bhargava, Brian K. Theisen, Ronald L. Hamilton, Adrian V. Lee, Adam M. Brufsky

**Affiliations:** ^1^ Division of Hematology, Oncology and Blood & Marrow Transplantation, University of Iowa Holden Comprehensive Cancer Center, University of Iowa Hospitals and Clinics, Iowa City, IA, USA; ^2^ Department of Pathology, University of Pittsburgh, Pittsburgh, PA, USA; ^3^ Division of Hematology and Oncology, University of Pittsburgh, Pittsburgh, PA, USA

**Keywords:** breast cancer, brain metastasis, high affinity folate receptor, folate receptor alpha, metastatic breast cancer

## Abstract

High affinity folate receptor (HFR) can be overexpressed in breast cancer and is associated with poor prognosis, however the expression in breast cancer brain metastases (BCBM) is unknown. The aim of this study was to analyze the rate of HFR expression in BCBM and its role in the prognosis of this high-risk cohort. We analyzed 19 brain metastasis (BM) and 13 primary tumors (PT) from a total of 25 patients. HFR status was assessed by immunohistochemistry. Median follow-up was 4.2 years (range 0.6-18.5). HFR was positive in 4/19 BM (21.1%) and in 1/13 PT (7.7%). Positive samples had low H-scores (range 1-50). 56% of patients had apocrine differentiation. OS was similar between patients with positive HFR (median OS 48 months) and negative HFR (median OS 69 months) (*P* = 0.25); and between patients with apocrine differentiation (median OS 63 months) and those without apocrine differentiation (median OS 69 months) (*P* = 0.49). To the best of our knowledge, this is the first analysis of HFR expression in BCBM. While previous studies associated the presence of HFR with worse prognosis; in our cohort HFR was positive in only 21.1% of BM with low levels of positivity. Neither HFR nor apocrine features had impact in OS.

## INTRODUCTION

Folic acid is required by proliferating cells for the synthesis of nucleotide bases and methylation of DNA and proteins among other metabolic tasks. To incorporate the folates, normal cells use low affinity reduced folate carriers (*K_m_* = 10^−5^ M) [1, 2]. The high affinity folate receptor (HFR) (*K_d_* = 10^−10^ M) is a membrane protein that is upregulated in several cancers of epithelial origin and rarely present in most normal cells [3, 4]. High expression of HFR has been documented in ovarian, breast, kidney, uterine and lung cancers [3–6].

The expression levels of HFR have been associated with disease stage, aggressiveness and survival in ovarian cancer and in non-small cell lung cancer [5, 7–10]. In breast cancer patients, HFR is overexpressed in 33% of primary tumors (PT) and this appeared to be associated with poor prognosis [11]. Analysis of the expression of HFR in triple-negative breast cancer (TNBC) has shown higher rates of positivity (50 – 80%) with worse prognosis for the HFR positive subgroup of patients [12–14].

Several conjugates of chemotherapy or immunotherapy with folate have been developed to target neoplastic cells that overexpress HFR [15–20]. Results of studies in ovarian cancer are promising, showing good tolerability and efficacy [21].

In patients with breast cancer, the development of brain metastasis (BM) represents a catastrophic event that results in poor prognosis and short survival [22]. New therapeutic approaches for this high risk group of patients are sorely needed. The HFR expression in breast cancer brain metastases (BCBM) is unknown. If the receptor is present, drugs that target HFR could become a novel approach to the treatment of BM.

The main aim of this study was to analyze the incidence of HFR expression in BCBM. Secondary aims were to correlate the incidence of HFR expression in BCBM with that of PT of the same patient and to evaluate its role in the prognosis.

## RESULTS

### Patient characteristics

Twenty-two of the 25 patients had complete clinical data and were included for the analysis of patient characteristics. Median age at the time of initial diagnosis of breast cancer was 51 years (range 24-74 years). Patient and tumor characteristics have been published as part of a larger clinical analysis elsewhere [22] and are summarized in Table 2. The majority of patients had stage II at diagnosis (45.3%), although patients of all stages were included; most patients were histologic grade 3 (59%). ER and HER2 were negative in 50% of cases, whereas PR was negative in 72.7% of patients. Approximately two thirds of patients received radiation therapy to the brain, with 4 patients receiving gamma knife, 3 patients receiving whole brain radiation therapy and 7 patients receiving the combination of the two. After the diagnosis of BM, patients received a median of 3 lines of chemotherapy (range 0 – 11).

The distribution of the different biological subtypes of breast cancer where as follows: ER+/HER2- 30%, ER+/HER2+ 15%, ER-/HER2+ 30% and ER-/HER2- 25%.

### Tissue analysis

**Figure 1:**
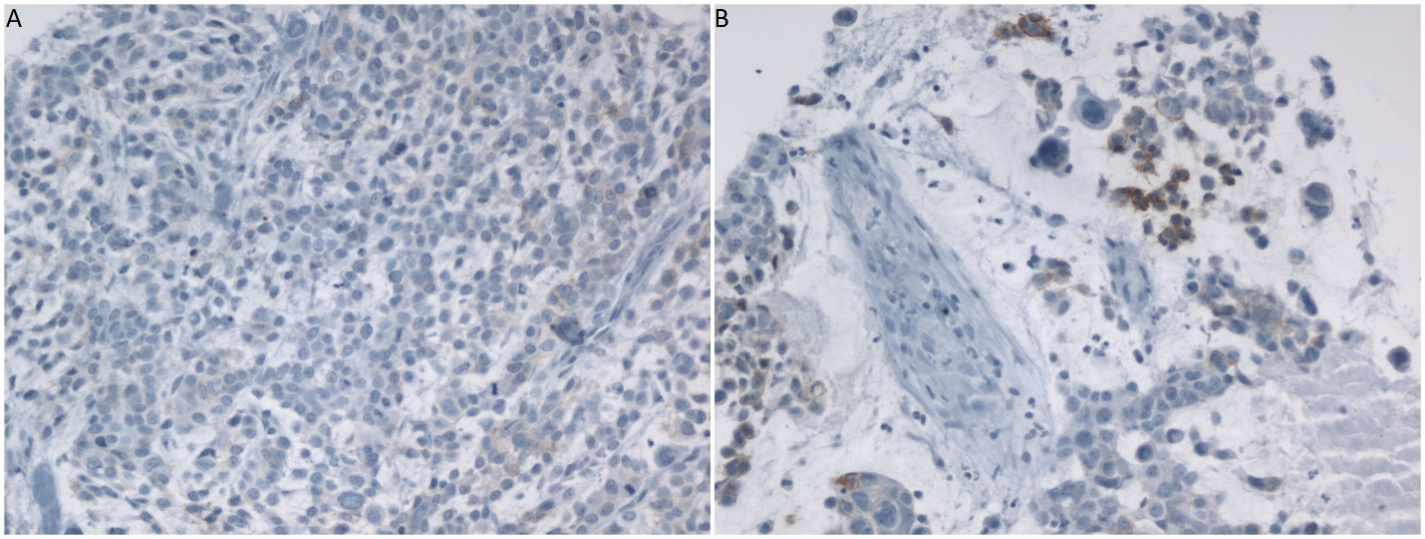
IHC staining for HFR on **A.** PT (H-score 10) and **B.** BM (H-score 15). Samples belong to the same patient.

**Figure 2:**
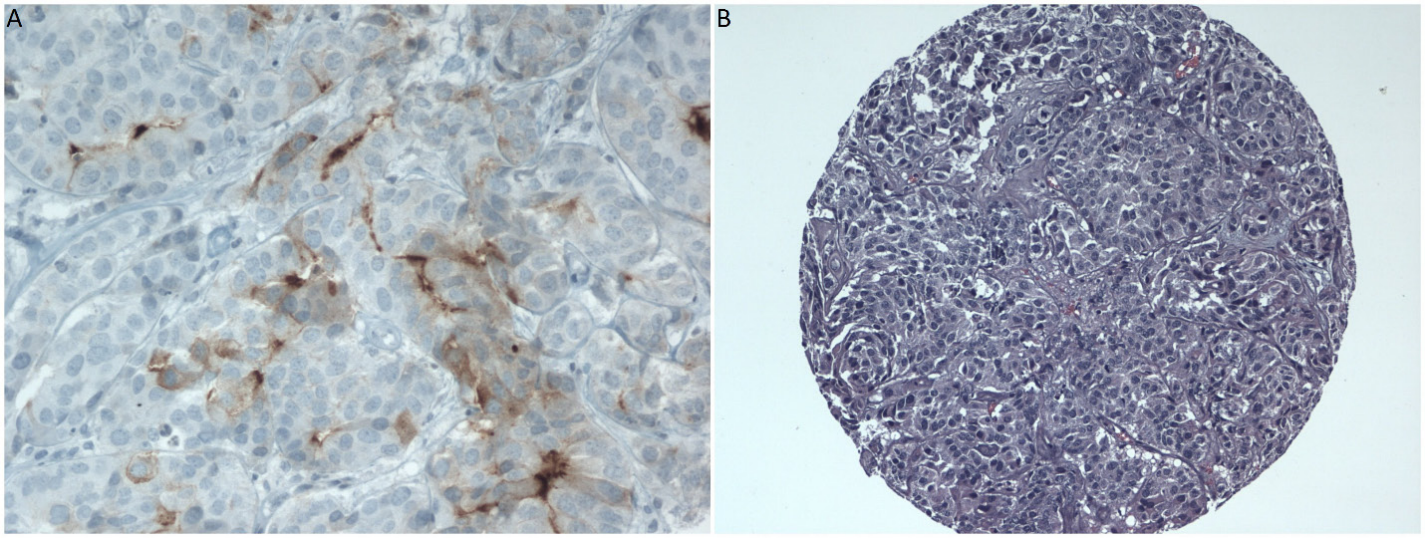
**A.** IHC staining for HFR on BM (H-score 50) and **B.** H&E of BM showing apocrine differentiation. Samples belong to the same patient.

A total of 25 patients were included in this study (Table 1). HFR was positive in 4/19 BM (21.1%) and in 1/13 PT (7.7%). Among the 4 patients with positive HFR in the BM, 1 patient had negative HFR in the PT, 1 patient had positive HFR in the PT and 2 patients had no PT tissue available. Figure 1 shows the IHC staining for the only patient who had positive HFR both in the PT and in the BM. The samples that were positive for HFR in our study had low levels of expression, with H-scores ranging from 1 to 50. Figure 2A shows the IHC staining for the BM sample that had the highest H-score of 50. Among the 4 patients with positive HFR in the BM, 1 patient had ER+ PR+ HER2 unknown breast cancer, 1 patient had ER- PR- HER2+ breast cancer and 2 patients had TNBC.

**Table 1:** Samples included in the study

Sample site	N
Primary tumor and brain metastasis from same patient	7
Brain metastasis only	12
Primary tumor only	6
Total	25

**Table 2:** Patient characteristics

	N	%*
**Histology**		
Ductal	18	81.8
Lobular	1	4.5
Mucinous	1	4.5
Medullary	1	4.5
Unknown	1	4.5
**Stage**		
1A	3	13.6
2A	6	27.2
2B	4	18.1
3A	1	4.5
3C	3	13.6
4	4	18.1
Unknown	1	4.5
**Grade**		
1	1	4.5
2	6	27.2
3	13	59
Unknown	2	9
**ER**		
Positive	11	50
Negative	11	50
**PR**		
Positive	6	27.2
Negative	16	72.7
**HER2**		
Positive	9	40.9
Negative	11	50
Unknown	2	9
**Radiation therapy**		
Yes	14	63.6
No	8	36.3
**Radiation type**		
Gamma knife	4	28.5
Whole brain	3	21.4
Gamma knife and whole brain	7	50
**Vital status**		
Alive	4	18.1
Dead	18	81.8

* Three patients had missing clinical data and were not included in the analysis of patient characteristics.

Evaluation of staining with H&E revealed that 11/19 BM (57.9%) and 7/13 PT (53.8%) had features consistent with apocrine differentiation. Overall, 14/25 patients (56%) had apocrine differentiation in either of their samples. Figure 2B shows the H&E staining of a BM sample that has apocrine differentiation.

### Survival analysis

After a median follow-up time of 4.2 years (range 0.6 – 18.5 years), 18.1% of patients are still alive. Median DFS was 29 months (95% CI: 22 months, 41 months), and median OS was 69 months (95% CI: 38 months, 93 months) for the entire cohort.

**Figure 3:**
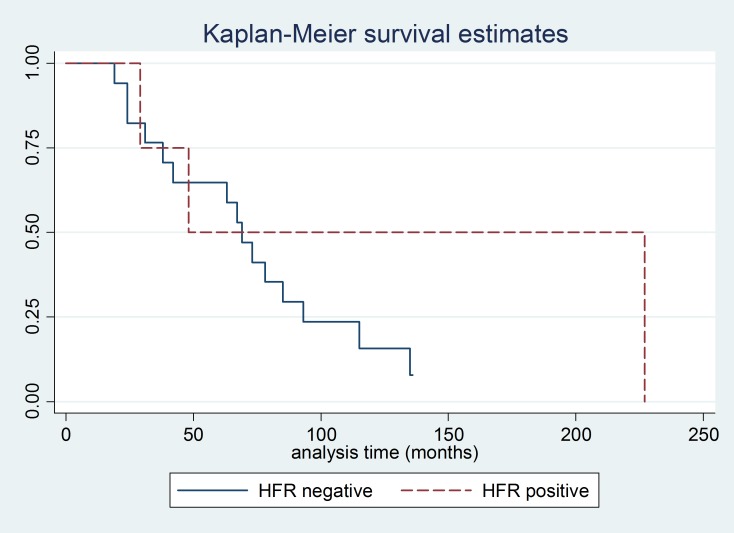
Kaplan Meier curve for overall survival according to HFR status

**Figure 4:**
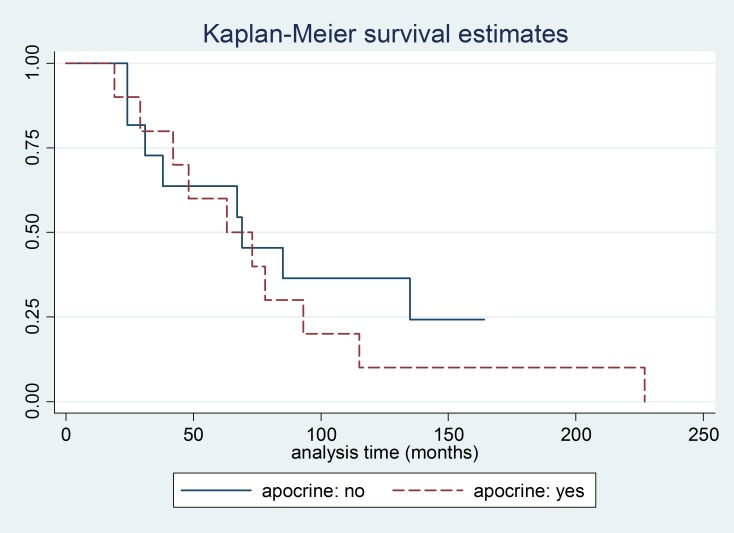
Kaplan Meier curve for overall survival according to apocrine differentiation

Analysis of OS showed a median of 48 months for the group of patients with positive HFR, as compared with a median of 69 months for the group of patients with negative HFR (*P* = 0.25) (Figure 3). Analysis of OS according to apocrine differentiation was also performed, this analysis showed that there was no difference in OS between patients with apocrine differentiation (median OS 63 months) and those without apocrine differentiation (median OS 69 months) (*P* = 0.49) (Figure 4).

We conducted an exploratory analysis of survival from the time of first tumor recurrence (either in brain or other sites) according to HFR status, the median for HFR negative patients was 30 months, whereas for HFR positive was 22 months (*P* = 0.36) (data not shown). Similarly, we analyzed survival from the date of diagnosis of BM according to HFR status, the median for HFR negative patients was 24 months, whereas for HFR positive was 12 months (*P* = 0.35) (data not shown).

## DISCUSSION

Previous studies have suggested a direct association between the presence of HFR in cancer cells and the development of a more aggressive tumor phenotype, including tumor growth and poor prognosis [7, 11, 13]. This has been hypothesized to be due to the increased intracellular folate levels in the malignant cells, which in turn facilitates most cellular metabolic processes.

Several clinical trials are evaluating different ways to target the HFR. Recently, a phase II trial in ovarian cancer showed that targeting the folate receptor with vintafolide, combined with chemotherapy, significantly improved progression-free survival compared with chemotherapy alone [21]. Phase III clinical trials of this and other agents are currently underway (NCT01170650 and NCT00849667).

Because of the potential for HFR as a target for treatment strategies and the fact that patients with BM are in substantial need for such novel therapies, we sought to analyze the incidence of HFR expression in BCBM and assess its prognostic role.

Our cohort was a high-risk group of patients who all developed BM. With a median age at diagnosis of breast cancer of 51 years, 45% of cases were HER2 positive and 25% were TNBC. Overall, we observed low rates of HFR expression. Only 21.1% of brain samples and 7.7% of PT were positive on IHC. These findings contrast with previous studies that have reported rates of HFR expression in breast cancer PT of around 22-33% [11–14]. These differences might be due to small sample sizes across studies. It is interesting to notice that in our study, the rate of HFR expression in BM was higher than in PT. The reason for this might be explained by the role of HFR in tumor aggressiveness [7], although this warrants further investigation.

In our analysis of H&E slides, we observed a high number of cases with apocrine differentiation, representing 56% of cases. Apocrine differentiation is a morphologic change in breast cancer, characterized by nuclear and cytoplasmic changes in the cells, that can be seen in up to 30% of all cases [23]. Similar to HFR overexpression, this morphologic change is associated with reduced ER and PR expression [13, 23–25]. In a recent study, patients with apocrine differentiation had similar DFS and OS compared with patients with invasive ductal carcinoma [26]. In our study, neither the presence of apocrine differentiation nor the expression of HFR had any impact in prognosis. Median OS for patients with negative HFR was longer than for patients with positive HFR, however the difference was not statistically significant.

We acknowledge that our study has some limitations. It is a retrospective analysis with a small number of samples, which could have affected our final results for the rates of HFR. We did not analyze the expression of HFR in other metastatic sites. However, despite these limitations, to the best of our knowledge this is the first study to report the incidence of HFR expression in BM from breast cancer and provides an insight into the correlation of expression between PT and distant metastasis.

In summary, our results showed that the rate and the intensity of HFR expression in breast cancer PT and BM are low. Around half of our patients had apocrine differentiation in their tumors. Neither the status of HFR nor the presence of apocrine features had impact in OS in our cohort. While this work requires validation in a larger cohort, these findings should prompt caution prior to conducting clinical trials in breast cancer patients with anti-HFR therapies.

## MATERIALS AND METHODS

### Tissue samples

We collected samples from 25 patients who underwent craniotomy with resection of BCBM at the University of Pittsburgh Medical Center. From these 25 patients, we had a total of 19 BM and 13 PT available in our tumor bank, which were included in this analysis. The number of patients with each tumor site is listed in Table 1. All samples were formalin fixed paraffin embedded (FFPE). Tissue microarrays (TMA) were built using a tissue arrayer (MTAI, Beecher Instruments, Inc, WI) to facilitate analysis of clinical samples using hematoxylin and eosin (H&E) and immunohistochemistry (IHC). H&E slides were used in order to take representative 0.6mm diameter cores of each tumor for analysis. Two cores were taken from each tumor. This study was approved by the University of Pittsburgh Institutional Review Board.

### Immunohistochemistry

IHC was performed using the Novocastra™ liquid mouse monoclonal Folate Receptor Alpha IgG1 antibody clone BN3.2 (Leica Biosystems, Newcastle Upon Tyne, UK) according to manufacturer's recommendation. Ovarian serous papillary carcinoma was used as the positive control. The staining for the HFR was scored using the H-score method (score range 0 – 300), which is the sum of the product of staining intensity (range 0 – 3) and percentage of stained cells (range 0 – 100). We documented the H-score for each available tumor site (PT or BM) independently for each patient.

### Clinical data

We used the clinical database of patients with BCBM from the University of Pittsburgh to collect the clinical information from the patients included in this study. Patients with leptomeningeal disease or dural metastases without parenchymal brain metastatic lesions were excluded from the study. Three patients had missing clinical data. Patients were diagnosed with breast cancer between April 1994 and May 2010. Information collected included age at diagnosis, demographic data, menopausal status, date of diagnosis, tumor histology and grade, stage at diagnosis, estrogen receptor (ER), progesterone receptor (PR) and human epidermal growth factor 2 (HER2) receptor status, dates and types of all treatments -including chemotherapy, hormonal therapies and targeted agents-, response to each treatment, survival status, date and location of distant metastasis, number of brain metastasis, and date of last follow-up or death.

### Statistical analysis

Patient and tumor characteristics are described by medians and frequencies. The staining score was dichotomized as positive (H-score = 1 or greater) or negative (H-score = 0). Disease free survival (DFS) was calculated as the time from diagnosis of breast cancer until the date of recurrence. Overall survival (OS) was the primary endpoint chosen to assess prognosis and was defined as the time from diagnosis of breast cancer until death from any cause or last follow-up for patients that were censored. Survival probabilities were estimated using the Kaplan Meier method. Log-Rank test analyzed differences in OS between groups. Alpha level of 0.05 was the cutoff of significance for all test statistics. Analysis was conducted using Stata 12.0 (College Station, TX).
